# Virucidal and antiviral activities of pomegranate (*Punica granatum*) extract against the mosquito-borne Mayaro virus

**DOI:** 10.1186/s13071-021-04955-4

**Published:** 2021-09-03

**Authors:** Tiago Souza Salles, Marcelo Damião Ferreira Meneses, Lucio Ayres Caldas, Thayane Encarnação Sá-Guimarães, Danielle M. de Oliveira, José A. Ventura, Renata Campos Azevedo, Ricardo M. Kuster, Márcia Regina Soares, Davis Fernandes Ferreira

**Affiliations:** 1 Institute of Microbiology, Federal University of Rio de Janeiro, Rio de Janeiro, Brazil; 2 Natural Products Research Institute, IPPN, Federal University of Rio de Janeiro, Rio de Janeiro, Brazil; 3 Capixaba Institute of Research, Technical Assistance and Rural Extension, Espirito Santo Vitoria, Brazil; 4 Chemistry Department, Federal University of Espírito Santo, Vitoria, Espirito Santo Brazil; 5 Institute of Chemistry, Federal University of Rio de Janeiro, Rio de Janeiro, Brazil; 6 National Institute of Science and Technology for Structural Biology and Bioimaging, INBEB, Rio de Janeiro, RJ Brazil; 7 Department of Molecular and Structural Biochemistry, North Carolina State University, Raleigh, NC USA; 8 Laboratory of Cellular Ultrastructure Hertha Meyer, Federal University of Rio de Janeiro, Rio de Janeiro, RJ Brazil

**Keywords:** Punicalagin, Biflavonoids, Alphavirus, Mayaro virus

## Abstract

**Background:**

The arthropod-borne Mayaro virus (MAYV) causes “Mayaro fever,” a disease of medical significance, primarily affecting individuals in permanent contact with forested areas in tropical South America. Recently, MAYV has attracted attention due to its likely urbanization. There are currently no licensed drugs against most mosquito-transmitted viruses. *Punica granatum* (pomegranate) fruits cultivated in Brazil have been subjected to phytochemical investigation for the identification and isolation of antiviral compounds. In the present study, we explored the antiviral activity of pomegranate extracts in Vero cells infected with Mayaro virus.

**Methods:**

The ethanol extract and punicalagin of pomegranate were extracted solely from the shell and purified by chromatographic fractionation, and were chemically identified using spectroscopic techniques. The cytotoxicity of the purified compounds was measured by the dye uptake assay, while their antiviral activity was evaluated by a virus yield inhibition assay.

**Results:**

Pomegranate ethanol extract (CC_50_ = 588.9, IC_50_ = 12.3) and a fraction containing punicalagin as major compound (CC_50_ = 441.5, IC_50_ = 28.2) were shown to have antiviral activity (SI 49 and 16, respectively) against Mayaro virus, an alphavirus. Immunofluorescence analysis showed the virucidal effect of pomegranate extract, and transmission electron microscopy (TEM) revealed damage in viral particles treated with this extract.

**Conclusions:**

The *P. granatum* extract is a promising source of antiviral compounds against the alphavirus MAYV and represents an excellent candidate for future studies with other enveloped RNA viruses.

**Graphical abstract:**

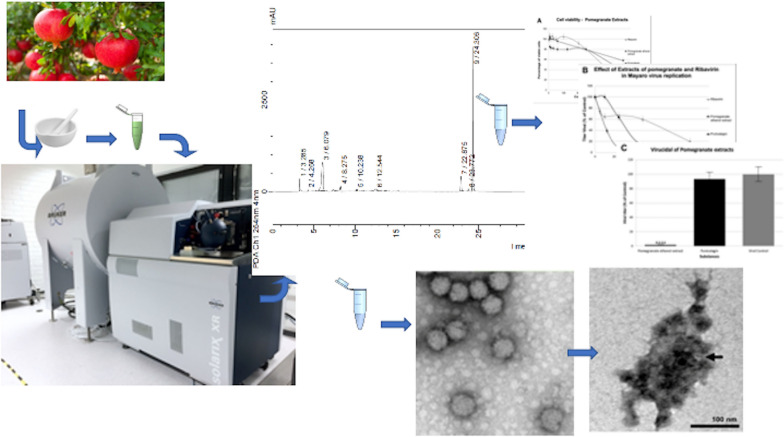

## Background

Mayaro fever is a dengue-like but usually non-fatal illness, occurring in tropical South America and endemic to the Amazon region [1] where outbreaks have been registered. The virus was first isolated in Trinidad and Tobago [2], and imported cases of human infection occur, but not frequently, outside the Amazon region [3], although some cases have been reported in other regions [4, 5].

Mayaro virus (MAYV) belongs to the *Togaviridae* family, *Alphavirus* genus, and is closely related to Chikungunya and other human alphaviruses [6]; it has two known genotypes (D and L), and is transmitted mainly by *Haemagogus janthinomys* mosquitoes [1]. Although MAYV has not been associated with fatal human disease, primary infections are often debilitating, with loss of productivity for weeks or even months due to severe arthralgia [7]. MAYV is an enveloped virus, and its genome is a single-strand, positive polarity RNA; the virus particle has an icosahedral symmetry and is 70 nm in size. Its genome has approximately 11 kilo-base pairs (kbp) coding for two polyproteins that are cleaved into non-structural proteins (nsP1, nsP2, snP3, and nsP4) and structural proteins (C, E2, E3, 6k, E1) [2].

Mayaro disease is characterized by mild or moderate fever of abrupt onset and short duration, chills, muscle and joint pain, and headache. Mosquitoes of *Haemagogus* spp. and *Aedes* spp. act as vectors of MAYV in rural and urban areas, respectively [8]. A recent finding shows the potential for MAYV transmission by the urban vectors *Aedes aegypti* and *Aedes albopictus*, thus contributing to the classification of the MAYV as an emerging virus, with the potential for inclusion in the urban cycle, as has occurred more recently with the Chikungunya virus [9–11].

The plant *Punica granatum*, commonly called pomegranate, is a deciduous shrub of the family *Lythraceae*, native to central and western Asia, and grows to 6–20 feet (less frequently to 30 feet) tall. It has long been cultivated for its orange-sized edible fruit and its attractive ornamental plant features [12], and has been used popularly as a medicinal plant since ancient times for several purposes. As an antiviral, pomegranate extracts are effective against herpes and influenza viruses. Furthermore, a topical micro-biocide could potentially be made for HIV prevention [13]. Punicalagin, the main ellagitannin from pomegranate fruits, targets and inactivates herpes simplex virus 1 (HSV-1) viral particles and can prevent binding, penetration, and cell-to-cell spread, as well as secondary infections [14]. A pomegranate polyphenol extract with punicalagin as a major compound was found to inhibit the influenza virus and had a synergistic effect with oseltamivir [15]. In the search for new biological properties associated with fruits cultivated in Brazil, we studied *P. granatum* for anti-MAYV activity.

## Methods

### Extract of *Punica granatum*

Fruits are produced in the state of Santa Catarina (latitude and longitude: −26.818664, −50.990292). Fruits (1.2 kg) were washed, halved, and washed again to extract all the juice and seeds, which were discarded. The shells were ground in a blender and extracted with EtOH—2L (PA, Vetec, Rio de Janeiro, Brazil) by maceration at room temperature for 15 days. Part (10 g) of the dried ethanol extract (42.7 g) was subjected to chromatography on cellulose acetate with a water/MeOH gradient (100% H_2_O, 9:1 → 1:9, 100% MeOH) as mobile phase to produce 34 fractions, which were analyzed by thin-layer chromatography (TLC) on cellulose (H_2_O:AcOH/9:1). Cellulose acetate, XAD-16, and Sephadex LH-20 were used as stationary phases for column chromatography. Those (23–25) with a large purple spot on the TLC plate sprayed with a sodium nitrite/acetic acid 10% solution were pooled and analyzed by high-performance liquid chromatography with diode array detection (HPLC/DAD), electrospray ionization/mass spectrometry (ESI/MS), and nuclear magnetic resonance (NMR).

### Structure analysis

Optical rotations were measured on a digital polarimeter. Circular dichroism spectra of biflavonoids were obtained with a Chirascan™ CD spectrometer (Applied Photophysics, UK). FT-ICR-ESI–MS spectra were obtained on a Bruker solariX model 9.4 (Bruker Daltonics, Bremen, Germany) mass spectrometer. An irregular C18 reversed-phase silica gel (RP-18, 5 μm, 20 × 5 mm) (Merck, Darmstadt, Germany) was used for analytical HPLC). ^1^H-NMR, APT, heteronuclear single quantum coherence (HSQC), and heteronuclear multiple-bond coherence (HMBC) NMR spectra in MeOD or DMSO as solvents and TMS as internal standard were recorded on Bruker DRX 400 and 500 MHz spectrometers (Germany).

### Cells and viruses

Vero cells from African green monkey kidney were purchased from the American Type Culture Collection (ATCC, Rockville, MD, USA) and grown in Dulbecco's modified Eagle medium (DMEM) supplemented with 2.5% fetal bovine serum, 0.23% NaHCO_3_ and antibiotics amphotericin B (25 µg/ml), 5 ml solution of penicillin (100 U/ml), and streptomycin (100 µg/ml). MAYV was obtained from the ATCC (VR-66, lineage TR 4675).

### Cytotoxicity assay

The cytotoxicity of compounds was determined using a technique called “dye uptake” [16], which consists of the incorporation of neutral red dye by living cells, followed by fixation with 20% formaldehyde. Subsequently, the dye was extracted with an extraction solution consisting of 50% methanol and 10% acetic acid in phosphate-buffered saline (PBS) and finally quantified with the plate reader (BIO-RAD model 3550) with a wavelength of 490 nm. Assays were performed on confluent monolayers of 96-well microplate-grown Vero cells (TPP, USA), which were treated with triplicate serial dilutions of the substances and kept in a greenhouse under appropriate conditions for 24 h. After this period, 100 µl of DMEM culture solution and neutral red at 50 µg/l were added to the cells. The plates were incubated for 3 h at 37 °C and 5% CO_2_. Next, cells were fixed for 20 min with 100 µl of 20% formaldehyde solution in PBS. The fixative solution was then removed, and the dye extracted for 20 min with 100 µl of the extraction solution (50% methanol and 1% acetic acid). Then, the plates were read in a spectrophotometer.

### Viral inhibition by extracts obtained from *P. granatum*

Confluent Vero cells in 24-well microplates were infected at multiplicity of infection (MOI) of 0.01 for 1 h. After adsorption, the culture medium was added with or without the substances at the indicated concentrations. After 24 h, the supernatant of cell cultures was collected for determination of the viral titer. Vero cells were cultured in 96-well microplates (TPP, USA). Supernatant collected from the infectivity inhibition experiments was added in serial dilutions to the cells. The tissue culture infective dose (TCID_50_) was calculated according to the Reed–Muench method [17]. The TCID_50_ experiments were performed in 96-well plates in quadruplicate and three independent experiments.

### Virucidal activity

For the virucidal activity, which is the effect of the substances directly on the virus particles, the substances were incubated 1:1 with 100 plaque-forming units (PFU) of purified MAYV. The virus/substance incubation period was either 1 h at 37 °C before the mixture was added to Vero cell monolayers, or the substance and virus were mixed immediately before adding to Vero cell monolayers. Confirmation of the virucidal effect was determined by plaque assay [18, 19]. We used confluent monolayers of Vero cells infected with MAYV. After 1 h adsorption, virus inoculum was removed, and monolayers were rinsed with PBS and incubated with growth medium with or without the different substances to be tested. Twenty-four hours post-infection, the cell culture supernatant was recovered and used for titration of extracellular infective virus. The latter was performed by plaque assay in Vero cells that had just reached confluence. The monolayers were overlaid with growth medium, supplemented with 10% FCS and 6% carboxymethylcellulose (CMC) (Sigma-Aldrich, St. Louis, MO, USA) and were further incubated in an atmosphere of 37 °C, 5% CO_2_ for 3 days. The monolayers were then stained with crystal violet (1%), and the virus plaques were counted [20].

### Immunofluorescence

For immunofluorescence analysis, infected Vero cells in 24-well plates were washed with PBS and fixed with 4% paraformaldehyde for 15 min at room temperature. Cells were permeabilized with 0.1% Triton X-100 in PBS for 10 min and then blocked for 15 min at 25 °C with a blocking solution that contained 3× rinsing with 3% BSA in PBS with fish gelatin (PBSA). Fixed cells were incubated for 2 h at 25 °C with the primary antibody (anti-HA) diluted in blocking solution. The secondary antibody (Cy3-conjugated anti-mouse IgG) was diluted in blocking solution and incubated with the cells for 1 h at 25 °C, followed by dilution with 546 phalloidin blocking solution (Invitrogen) for 40 min at 25 °C. The cells were washed five times with PBSA after each antibody treatment. Finally, cells were treated with ProLong Gold reagent with DAPI (Invitrogen). Fluorescence was analyzed microscopically with an automated fluorescence microscope (Olympus, Tokyo, Japan), and the cells were photographed with an Olympus DP70 digital camera (Tokyo, Japan).

### Transmission electron microscopy (TEM)

MAYV was purified by ultra-centrifugation in a tartrate two-step gradient (15% and 35%) at 24,000 rpm (sw-28 rotor) in a Beckman ultracentrifuge. The resulting virus band was collected and incubated with the test substances Pomegranate ethanol extract for 1 h at 37 °C. Untreated purified MAYV was used as control. The viruses were then placed on 400-mesh carbon grids for 1 min. Then the grids were washed three times and contrasted with 1% uranyl acetate. Finally, the material was visualized under a transmission electron microscope [16].

### Statistical analysis

The selectivity index (SI) was determined by the ratio of CC_50_ to IC_50_. All experiments were performed in triplicate, and three independent experiments were conducted. Data were presented as mean ± SD, and the *t*-test was used to evaluate the difference between the test and control. One-way ANOVA and Dunnett’s multiple comparisons test were performed with GraphPad Prism 7 (GraphPad Software). All significant values had *P*-values less than 0.05.

## Results

### Structure identification of *P. granatum* isolate

The chromatographic fractionation of the EtOH extract fruits led to isolation of 34 fractions, which were analyzed by TLC. Those (23–25) with a large purple spot on the TLC plate sprayed with a sodium nitrite/acetic acid 10% solution were pooled and analyzed by HPLC/DAD, ESI/MS, and NMR.

**Fig. 1:**
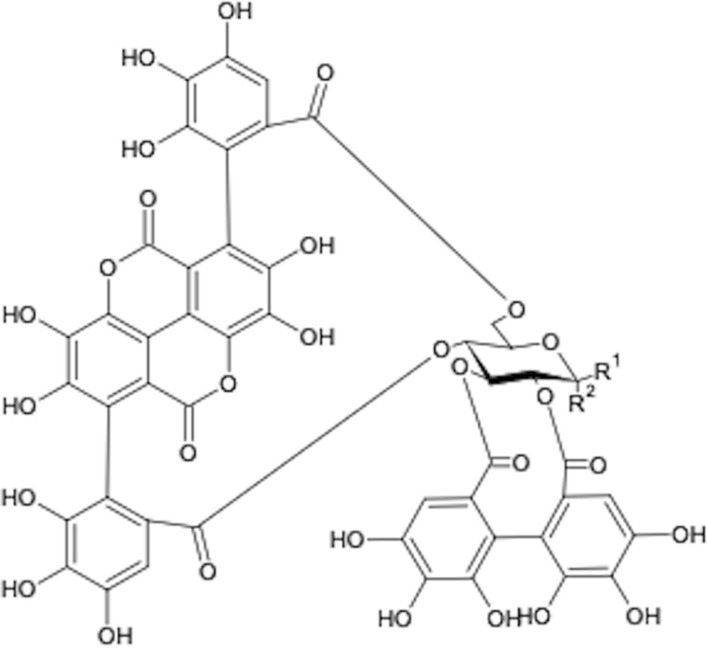
Punicalagin α and β. Chemical structure of anomers (α and β), α-punicalagin, R1 = H and R2 = OH; β-punicalagin, R1 = OH and R2 = H

Two major peaks at 20.9 min (237, 276, and 390 nm) and 22.1 min (232, 276, and 393 nm) were observed on HPLC/DAD chromatograms. ESI–MS spectrum of this sample furnished a pseudo-molecular ion [M−H+]^−^ 1083. The NMR data are in accordance with the two anomers (α and β) of punicalagin (Fig. 1), a typical ellagitannin of *P. granatum* pericarp. Furthermore, α-punicalagin anomeric hydrogen was detected on 1H-NMR as a doublet at 5.12 ppm (*J* = 3.74 Hz) and a doublet at 4.51 ppm (*J* = 7.87 Hz) for β-punicalagin (Fig. 1). The HSQC spectrum showed 92.28 and 96.30 for the anomeric carbons, respectively. Ellagic acid [M−H+]^−^ 301, gallagic acid [M−H+]^−^ 601 and punicalin [M−H+]^−^ 781 were identified in the isolate as minor compounds. Interestingly, cellulose acetate was used for the first time as stationary phase for column chromatography of ellagitannins, and the sample with α- and β-punicalagins as major compounds was obtained with a unique chromatographic run.

### Cytotoxicity

**Fig. 2:**
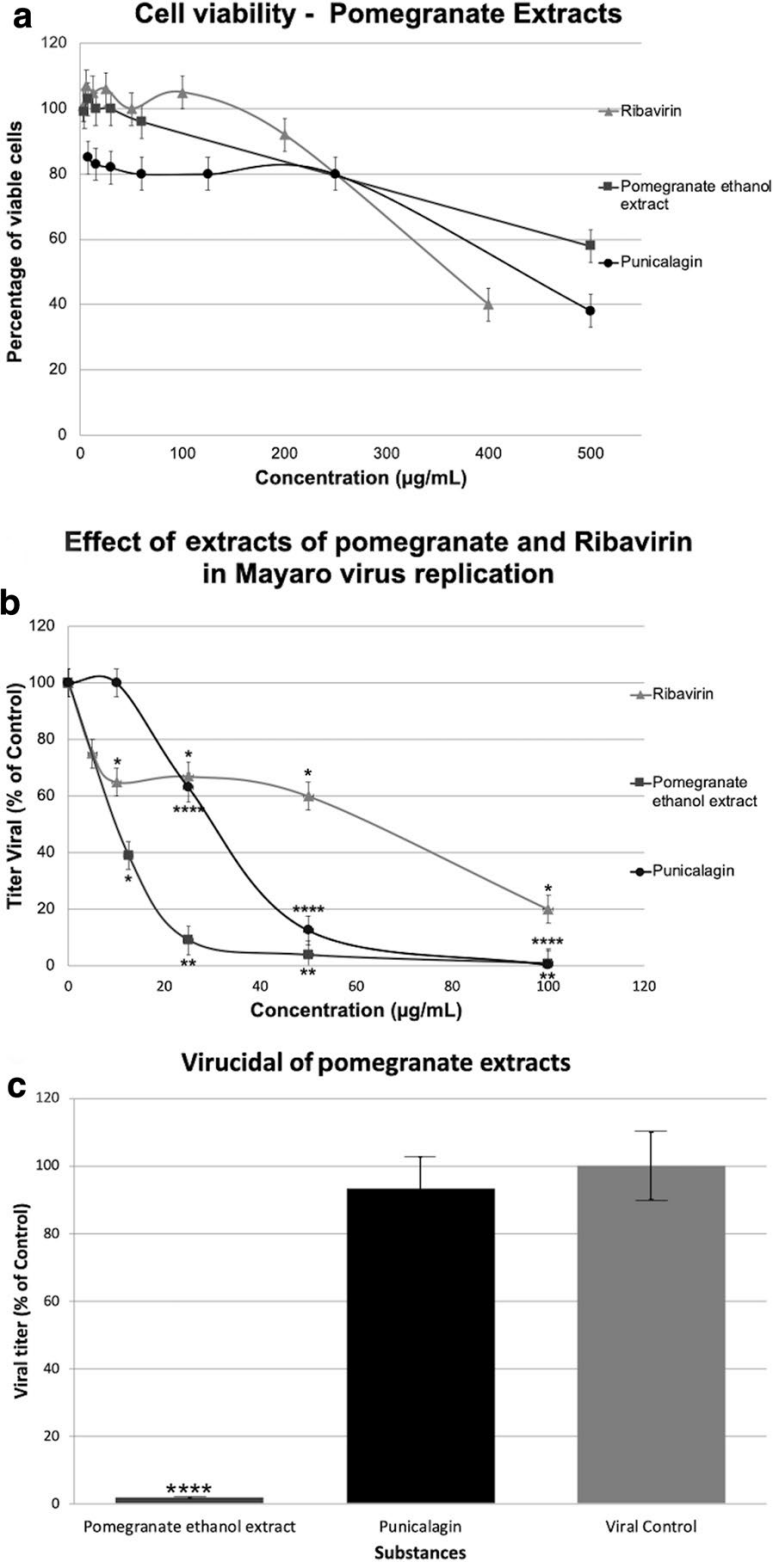
**a** Cytotoxic effect: the cytotoxicity of the substances was determined by the dye uptake technique, which consists of the incorporation of neutral red dye by living cells, where the substances were applied in an initial concentration of 2 mg/ml, and then serial dilutions were made up to the last well of the column in the 96-well plates. The values obtained in the reading were transformed into percentage of viable cells in each concentration of the cells treated with the substances compared to the control cells (untreated). The lead gray curve represents punicalagin, black represents ethanol extract of pomegranate, and gray represents ribavirin. *y*-axis, percentage of viable cells (Vero); *x*-axis, different concentrations of the substances. **b** Antiviral activity: confluent Vero cells were infected with a multiplicity of infection (MOI) of 0.1 for 1 h. After adsorption, culture medium was added without the substances in the viral control and with the substances at the concentrations indicated in the experiment. After 24 h, the supernatant was collected and titrated by the TCID_50_ titration method. The lead gray curve represents punicalagin, black represents ethanol extract of pomegranate, and gray represents ribavirin. *y*-axis, virus titer; *x*-axis, the different concentrations of the substances. One-way analysis of variance (ANOVA) followed by Dunnett’s multiple comparisons test (*P* < 0.05) was used in the capture experiments, and Dunnett’s test (*P < 0.05; **P < 0.01; ****P < 0.0001) was used to compare the viral inhibition between the control viral and other concentrations of ethanol extract from pomegranate and punicalagin. Cells were treated with 12.5, 25, 50, and 100 µg/ml of ethanol extract of pomegranate and punicalagin at 24 h post-infection. The *y*-axis bar graph shows the viral titer in % of control ; the *x*-axis shows the concentrations of extract on the left and the different ethanol extracts of pomegranate concentrations on the right, 12.5, 25, 50, and 100 µg/ml One-way analysis of variance (ANOVA) followed by Dunnett's multiple comparison test (*P* < 0.05) was used in the capture experiments, and Dunnett’s test (*P < 0.05) was used to compare the viral inhibition between the control viral with other concentrations of ribavirin. Cells were treated with 12.5, 25, 5 0, and 100 µg/ml of ethanol extract of pomegranate at 24 h post-infection. The *y*-axis bar graph shows the viral titer in % of control; the *x*-axis shows the concentrations of extract on the left and the different extracts of pomegranate concentrations on the right, 25, 50, 100, 200, 400 µg/ml. **c** Virucidal activity: in order to evaluate whether the tested substances have a “virucidal” effect, that is, they interact directly with the viral particle, the substances (at the concentration of twice the value that obtained the best activity) were incubated with the virus at a final concentration of approximately 100 PFU/ml for 1 h at 37 °C. Soon after this treatment, this inoculum was titrated by plaque assay. Vero cell monolayers were treated with the substances at a concentration of 200 µg/ml: *x*-axis, the substances, ethanol extract of pomegranate, punicalagin, and viral control (VC). *y*-axis, percentage of virus titer. One-way analysis of variance (ANOVA) followed by Dunnett’s multiple comparison test (*P* < 0.05) was used in the capture experiments, and Dunnett’s test (****P < 0.0001) was used to compare the viral inhibition between control viral with other concentrations of ethanol extract of pomegranate and punicalagin

The cytotoxicity of the compounds was determined by “dye uptake” [15, 21–30], which consists of the incorporation of neutral red dye by living cells and subsequent extraction and quantification by a spectrophotometer at a wavelength of 490 nm (Fig. 2a), to which it is possible to determine a viable concentration to be used in the antiviral test, and the data were used to determine the CC_50_ (Table 1). The antiviral activity of the compounds was evaluated as the ability of the substances to inhibit MAYV replication in Vero cells at a nontoxic concentration (Fig. 2b).

**Table 1:** Cytotoxicity and anti-MAYV activity of punicalagin and ethanol extract of pomegranate

Substance	CC_50_^a^	IC_50_^b^	IS_50_^c^	RP^d^
Pomegranate ethanol extract	590.8 ± 17.7	12.3 ± 0.4	48	6
Punicalagin	425 ± 12.8	29.9 ± 0.9	14	1.75
Ribavirin	523.1 ± 10.5	62.5 ± 4.4	8	–

^a^50% cytotoxic concentration

^b^50% inhibitory concentration of viral replication

^c^Selectivity index = CC50/IC50

^d^Relative potency = IS_50_(substance)/IS_50_(ribavirin)

### Antiviral activity

Vero cells infected with MAYV were treated with different concentrations of the compounds. All the compounds exhibited strong antiviral activity (IC_50_) when compared to ribavirin, a known antiviral used in the treatment of hepatitis C, respiratory syncytial virus, and other viral infections. The antiviral activity was dose-dependent and reached values above 95% inhibition at the highest nontoxic concentrations tested (CC_50_). The selectivity index (SI) was determined by the ratio of CC_50_ to IC_50_. All experiments were performed in triplicate, and three independent experiments were conducted. Data are presented as mean ± SD, and *t*-tests were used to evaluate the difference between the test and control. A *P*-value of < 0.05 was considered statistically significant (Table 1).

### Virucidal activity

For the virucidal activity, we used confluent monolayers of Vero cells infected with MAYV that had been exposed to the substances according to various methods. The pomegranate ethanol extract showed 98% virucidal activity on MAYV particles, whereas punicalagin did not show detectable virucidal activity (Fig. 2c).

**Fig. 3:**
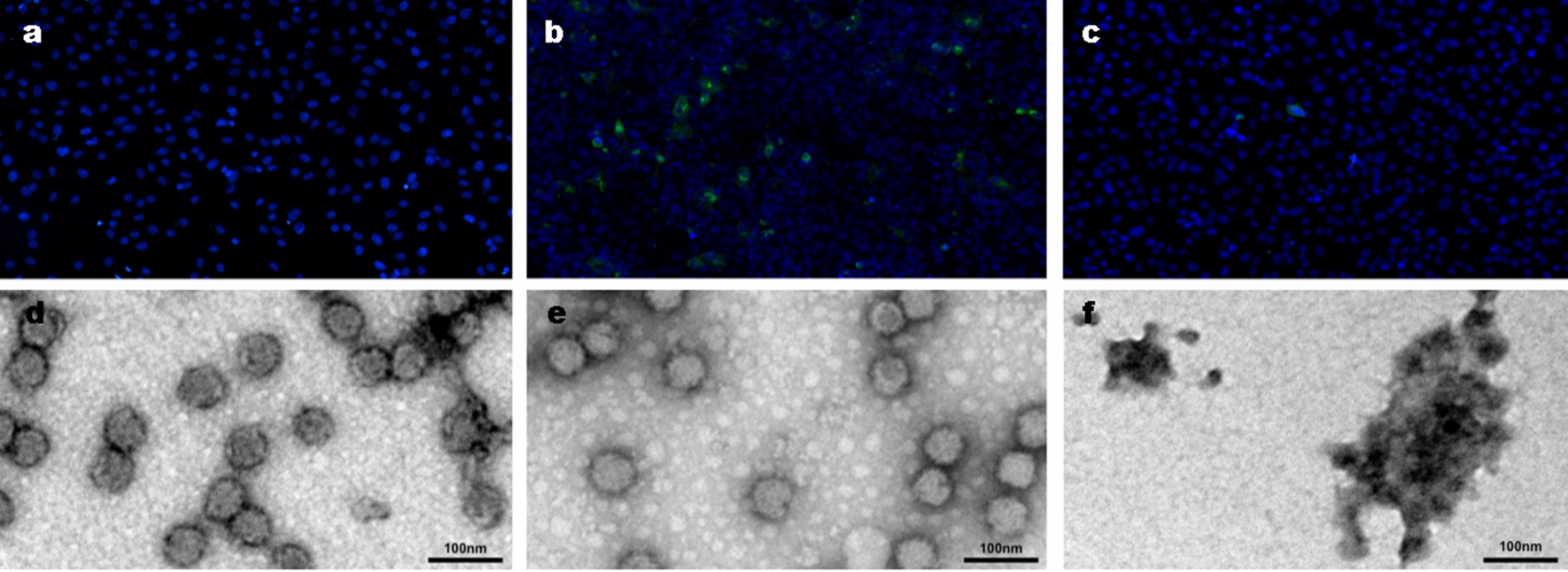
Immunofluorescence image: cells were treated with ethanol extract of pomegranate along with MAYV according to the virucidal experiment. As already noted, the ethanol extract of pomegranate had a significant positive effect on virus replication: **a** Cell control, **b** virus control, **c** ethanol extract of pomegranate. Nuclear staining in blue with DAPI and green with Sybr Green anti-mayaro IgG antibody. Transmission electron microscopy: ethanol extract of pomegranate was used at a concentration of 200 µg/ml with a suspension containing purified MAYV and then incubated for 1 h at 37 °C. The sample was placed on a 300-mesh carbon film grid then counterstained with uranyl acetate. Subsequently, it was analyzed in the electron microscope: **d** purified virus, **e** virus control, **f** ethanol extract of pomegranate. Image measurement bar is 100 nm in length

We also confirmed virucidal activity by immunofluorescence microscopy (IFM) and TEM. Pomegranate ethanol extract at a concentration of 200 μg/ml was mixed with a MAYV suspension (moi = 0.2) or cell media as control and incubated for 1 h at 37 °C. We observed a reduction in cell fluorescence when we infected the cells with virus-treated particles when compared to the viral control (Fig. 3b, c). A similar experiment was performed for TEM. Purified MAYV was mixed with pomegranate ethanol extract (200 μg/ml) or cell media (control) and then incubated for 1 h at 37 °C. In Fig. 3d, we can see damaged virus particles, apparently fused together with other virus particles as indicated by arrows (Fig. 3f).

## Discussion

The virus is a unique pathogen that is incapable of replicating without the host cell. Cell metabolism and cellular machinery are important for viral replication. Therefore, a bioactive substance that attacks virus replication will often have adverse effects on the host cell as well. In vitro antiviral studies (screening) are important for the evaluation of the safety, efficacy, and identification of the mechanism of action before such substances can be further tested in animals and humans [21, 30]. When a culture system in which the virus undergoes a complete viral replication cycle is available, it is possible to investigate the antiviral activity and generate clear data, such as the magnitude of inhibition, the steps of the virus replication cycle affected by the prospective drug, and evaluation of cytotoxicity to vertebrate cells, among other approaches [21]. In the case of natural compounds, there is an increasing number of potentially useful plants and herbs that need to be exploited, and their therapeutic applications are important weapons to be used against most virus families, and even for emerging viruses. The in vitro studies of antiviral effect, therefore, provide the relevant information necessary and the concentrations of the substances to be used for further tests, before they can that go to clinical trials, making it possible to access the risk–benefit [3, 18, 19, 27, 31–34].

Our results indicate that all tested substances have potential antiviral activity in concentrations that are nontoxic for Vero cells. According to our knowledge, this is the first report of antiviral activity for the substances pomegranate ethanol extract and punicalagin against an alphavirus [14, 23, 35]. The pomegranate ethanol extract showed a selectivity index value of 49, and the fraction containing punicalagin as the main component had a selectivity index of 15, as shown in Table 1. Surprisingly, this substance was previously described with antiviral activity against other viruses, such as HSV-1 and HSV-2 and influenza A and B [13, 15, 24, 29, 36–38]. Antiviral activity of several parts of this plant has been reported for HSV, influenza, respiratory syncytial virus, and HIV [13, 15, 24, 29, 36–38]. Given that this plant can inhibit the replication of viruses from different families, we understand that components from this plant may have different mechanisms of action against virus replication, including virucidal effects for some viruses. Our results show that these substances of pomegranate ethanol extract and punicalagin have antiviral and virucidal activity against an alphavirus and are potent candidates for further studies.

## Conclusions

In summary, the *P. granatum* ethanol extract showed a high selectivity index value of 49 and significant virucidal activity of approximately 98%, while the fraction containing punicalagin as the main component had a selectivity index of 15, with strong antiviral activity. The fact that this plant has great potential as an antiviral and can inhibit the replication of viruses from different families with different replication strategies is very important and suggests multiple targets of action. Our results show that these substances have good antiviral activity against the alphavirus MAYV and are excellent candidates for future studies with other enveloped RNA viruses.

## Data Availability

Not applicable.

## Abbreviations

ATCC: American Type Culture Collection
CC_50_: 50% Cytotoxic concentration
MAYV: Mayaro virus
CMC: Carboxymethylcellulose
HSV-1: Herpes simplex virus 1
HSV-2: Herpes simplex virus 2
HIV: Human immunodeficiency virus
IC_50_: 50% inhibitory concentration
IFM: Immunofluorescence microscopy
MOI: Multiplicity of infection
PBS: Phosphate-buffered saline
PFU: Plaque-forming unit
RP: Relative potency
SI: Selectivity index
TLC: Thin-layer chromatography
TCID_50_: 50% tissue culture infective dose
TEM: Transmission electron microscopy

## References

[1] Azevedo RSS, Silva EVP, Carvalho VL, Rodrigues SG, Neto JPN, Monteiro HAO (2009). Mayaro fever virus, Brazilian Amazon. Emerg Infect Dis.

[2] Mezencio JMS, de Souza W, Fonseca MEF, Rebello MA (1990). Ultrastructural study of Mayaro virus replication in BHK-21 cells. Arch Virol.

[3] Coimbra TLM, Santos CLS, Suzuki A, Petrella SMC, Bisordi I, Nagamori AH (2007). Mayaro virus: imported cases of human infection in São Paulo state, Brazil. Rev Inst Med Trop Sao Paulo.

[4] Zuchi N, Heinen L, Santos M, Pereira F, Dezengrini SR (2014). Molecular detection of Mayaro virus during a dengue outbreak in the state of Mato Grosso, Central-West Brazil. Mem Inst Oswaldo Cruz.

[5] Maia LMS, Bezerra MCF, Costa MCS, Souza EM, Oliveira MEB, Ribeiro ALM (2019). Natural vertical infection by dengue virus serotype 4, Zika virus and Mayaro virus in *Aedes* (*Stegomyia*) *aegypti* and *Aedes* (*Stegomyia*) *albopictus*. Med Vet Entomol.

[6] Strauss JH, Strauss EG (1994). The alphaviruses: gene expression, replication, and evolution. Microbiol Rev.

[7] Weaver SC, Reisen WK (2010). Present and future arboviral threats. Antiviral Res.

[8] Figueiredo LTM, Figueiredo LTM (2016). Serious disease outbreaks caused by viruses transmitted by *Aedes aegypti* in Brazil. Rev Soc Bras Med Trop.

[9] Long KC, Ziegler SA, Thangamani S, Hausser NL, Kochel TJ, Higgs S (2011). Experimental transmission of Mayaro virus by *Aedes aegypti*. Am J Trop Med Hyg.

[10] de Figueiredo MLG, Figueiredo LTM, de Figueiredo MLG, Figueiredo LTM (2014). Emerging alphaviruses in the Americas: Chikungunya and Mayaro. Rev Soc Bras Med Trop.

[11] Wiggins K, Eastmond B, Alto BW (2018). Transmission potential of Mayaro virus in Florida *Aedes aegypti* and *Aedes albopictus* mosquitoes. Med Vet Entomol.

[12] Missouri botanical garden. Missouri botanical garden. *Punica granatum*. http://www.missouribotanicalgarden.org/PlantFinder/PlantFinderDetails.aspx?taxonid=286059&isprofile=0&letter=P. Accessed 15 June 2021.

[13] Arun N, Singh DP (2012). *Punica granatum*: a review on pharmacological and therapeutic properties. Int J Pharm Sci Res.

[14] Lin Y-M, Flavin MT, Schure R, Chen F-C, Sidwell R, Barnard DI (1999). Antiviral activities of biflavonoids. Planta Med.

[15] Haidari M, Ali M, Ward Casscells S, Madjid M (2009). Pomegranate (*Punica granatum*) purified polyphenol extract inhibits influenza virus and has a synergistic effect with oseltamivir. Phytomedicine.

[16] Borenfreund E, Puerner JA (1985). Toxicity determined in vitro by morphological alterations and neutral red absorption. Toxicol Lett.

[17] Reed LJ, Muench H (1938). A simple method of estimating fifty per cent endpoint. Am J Epidemiol.

[18] Pujol CA, Ray S, Ray B, Damonte EB (2012). Antiviral activity against dengue virus of diverse classes of algal sulfated polysaccharides. Int J Biol Macromol.

[19] Uozaki M, Yamasaki H, Katsuyama Y, Higuchi M, Higuti T, Koyama AH (2007). Antiviral effect of octyl gallate against DNA and RNA viruses. Antivir Res.

[20] Reynolds ES (1963). The use of lead citrate at high pH as an electron-opaque stain in electron microscopy. J cell biol.

[21] Orhan DD, Özçelik B, Özgen S, Ergun F (2010). Antibacterial, antifungal, and antiviral activities of some flavonoids. Microbiol Res.

[22] Seeram N, Adams L, Henning S, Niu Y, Zhang Y, Nair M (2005). In vitro antiproliferative, apoptotic and antioxidant activities of punicalagin, ellagic acid and a total pomegranate tannin extract are enhanced in combination with other polyphenols as found in pomegranate juice. J nutr biochem.

[23] Lin L-T, Chen T-Y, Chung C-Y, Noyce RS, Grindley TB, McCormick C (2011). Hydrolyzable tannins (chebulagic acid and punicalagin) target viral glycoprotein-glycosaminoglycan interactions to inhibit herpes simplex virus 1 entry and cell-to-cell spread. J Virol.

[24] de Melo Júnior EJM, Raposo MJ, Lisboaneto JA, Diniz MFA, Marcelino Júnior CAC, Sant’Ana AEG (2002). Medicinal plants in the healing of dry socket in rats: microbiological and microscopic analysis. Phytomedicine.

[25] Li J, Huang H, Feng M, Zhou W, Shi X, Zhou P (2008). In vitro and in vivo anti-hepatitis B virus activities of a plant extract from *Geranium carolinianum* L.. Antivir Res.

[26] Weniger B, Vonthron-Sénécheau C, Kaiser M, Brun R, Anton R (2006). Comparative antiplasmodial, leishmanicidal and antitrypanosomal activities of several biflavonoids. Phytomedicine.

[27] Adnan A, Allaudin ZN, Hani H, Loh H-S, Khoo T-J, Ting KN (2019). Virucidal activity of *Garcinia parvifolia* leaf extracts in animal cell culture. Complement Altern Med.

[28] Covington CL, Junior FMS, Silva JHS, Kuster RM, de Amorim MB, Polavarapu PL (2016). Atropoisomerism in biflavones: the absolute configuration of (−)-agathisflavone via chiroptical spectroscopy. J Nat Prod.

[29] Wannan BS, Waterhouse JT, Gadek PA, Quinn CJ (1985). Biflavonyls and the affinities of Blepharocarya. Biochem Syst Ecol.

[30] Jeong HJ, Ryu YB, Park SJ, Kim JH, Kwon HJ, Kim JH (2009). Neuraminidase inhibitory activities of flavonols isolated from *Rhodiola rosea* roots and their in vitro anti-influenza viral activities. Bioorg Med Chem.

[31] Spindola KCW, Simas NK, Salles TS, de Meneses MDF, Sato A, Ferreira D (2014). Anti-Mayaro virus activity of *Cassia australis* extracts (Fabaceae, Leguminosae). Parasites Vectors.

[32] dos Santos AE, Kuster RM, Yamamoto KA, Salles TS, Campos R, de Meneses MDF (2014). Quercetin and quercetin 3-*O*-glycosides from *Bauhinia longifolia* (Bong.) Steud. show anti-Mayaro virus activity. Parasites Vectors.

[33] Ferraz AC, Moraes TDFS, da Cruz Nizer WS, dos Santos M, Tótola AH, Ferreira JMS (2019). Virucidal activity of proanthocyanidin against Mayaro virus. Antivir Res.

[34] Camini FC, da Silva TF, da Silva Caetano CC, Almeida LT, Ferraz AC, Alves Vitoreti VM (2018). Antiviral activity of silymarin against Mayaro virus and protective effect in virus-induced oxidative stress. Antivir Res.

[35] Araújo M, Santos C, Cavalcanti J, Pereira F, Mendes G, Werle A (2011). Proposed active compounds from *Ouratea parviflora*. J Med Plant Res.

[36] Zakaryan H, Arabyan E, Oo A, Zandi K (2017). Flavonoids: promising natural compounds against viral infections. Arch Virol.

[37] de Lima MRF, de Souza Luna J, dos Santos AF, de Andrade MCC, Sant’Ana AEG, Genet JP (2006). Anti-bacterial activity of some Brazilian medicinal plants. J Ethnopharmacol.

[38] Seeram NP, Heber D, Schulman RN (2006). Pomegranates: ancient roots to modern medicine.

